# Feasibility, acceptability and potential sustainability of a ‘diagonal’ approach to health services for female sex workers in Mozambique

**DOI:** 10.1186/s12913-018-3555-2

**Published:** 2018-10-03

**Authors:** Yves Lafort, Malica Sofia Ismael de Melo, Faustino Lessitala, Sally Griffin, Matthew Chersich, Wim Delva

**Affiliations:** 1 International Centre for Reproductive Health, Ghent University, Ghent, Belgium; 2 International Centre for Reproductive Health-Mozambique, Maputo, Mozambique; 3 Wits Reproductive Health and HIV Institute, Faculty of Health Sciences, University of the Witwatersrand, Johannesburg, South Africa; 4 The South African DST-NRF Centre of Excellence in Epidemiological Modelling and Analysis (SACEMA), University of Stellenbosch, Stellenbosch, South Africa; 5 Center for Statistics, Hasselt University, Diepenbeek, Belgium; 6 Rega Institute for Medical Research, KU Leuven, Leuven, Belgium

**Keywords:** Sexual and reproductive health, Female sex workers, Service delivery, Implementation research, Mixed methods, Mozambique

## Abstract

**Background:**

Female sex workers (FSWs) in many settings have restricted access to sexual and reproductive health (SRH) services. We therefore conducted an implementation study to test a ‘diagonal’ intervention which combined strengthening of FSW-targeted services (vertical) with making public health facilities more FSW-friendly (horizontal). We piloted it over 18 months and then assessed its performance.

**Methods:**

Applying a convergent parallel mixed-methods design, we triangulated the results of the analysis of process indicators, semi-structured interviews with policy makers and health managers, structured interviews with health care providers and group discussions with peer outreach workers. We then formulated integrated conclusions on the interventions’ feasibility, acceptability by providers, managers and policy makers, and potential sustainability.

**Results:**

The intervention, as designed, was considered theoretically feasible by all informants, but in practice the expansion of some of the targeted services was hampered by insufficient financial resources, institutional capacity and buy-in from local government and private partners, and could not be fully actualised. In terms of acceptability, there was broad consensus on the need to ensure FSWs have access to SRH services, but not on how this might be achieved. Targeted clinical services were no longer endorsed by national government, which now prefers a strategy of making public services more friendly for key populations. Stakeholders judged that the piloted model was not fully sustainable, nor replicable elsewhere in the country, given its dependency on short-term project-based funding, lack of government endorsement for targeted clinical services, and viewing the provision of community activities as a responsibility of civil society.

**Conclusions:**

In the current Mozambican context, a ‘diagonal’ approach to ensure adequate access to sexual and reproductive health care for female sex workers is not fully feasible, acceptable or sustainable, because of insufficient resources and lack of endorsement by national policy makers for the targeted, vertical component.

## Background

In many settings, female sex workers (FSWs) are amongst the most vulnerable groups for adverse sexual and reproductive health outcomes. They are 10 times more likely than other women to acquire HIV [1], and in countries with medium and high background HIV epidemics the overall prevalence was estimated to be 30.7% in 2012 [2]. Other sexually transmitted infections (STI), including HPV [3, 4], unintended pregnancies [5–7] and sexual violence [8] are also very common among FSWs. At the same time, their access to prevention and care services is severely restricted. They are often migrants, working in unfamiliar environments [9]. At public health services they are confronted with stigmatising attitudes by providers and other users [10, 11]. They therefore often avoid these services, or hide their profession when they do, resulting in care not being tailored to their needs [12]. In addition, the morning opening hours of clinics are not compatible with their nightly work [13]. For these reasons, several initiatives have developed services specifically targeted to sex workers, either through mobile outreach or parallel stand-alone clinics [14–16]. These initiatives, however, generally achieve a low coverage and offer a limited scope of services [17].

**Fig. 1:**
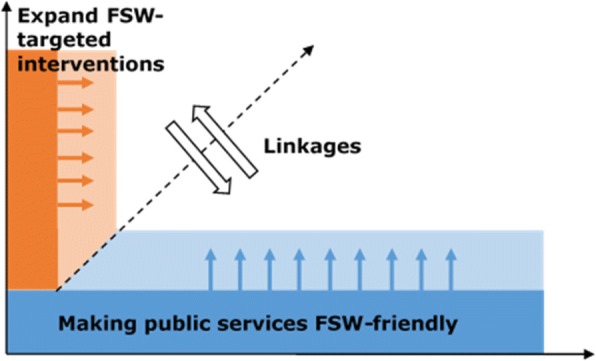
A ‘diagonal’ approach to enhance access to health services for high-risk women

Our hypothesis for this study was that, to ensure a comprehensive package of SRH services for FSWs, a ‘diagonal’ approach is needed, combining FSW-targeted services (vertical) with improved access to general health services (horizontal) (Fig. 1). Targeted interventions have demonstrated to be effective in increasing uptake of basic SRH commodities and services, such as peer education, condoms, lubricants, simple contraceptive methods, for example oral contraception, STI care and HIV testing, and in mobilising and empowering the FSW community [15, 18–21]. They, however, rarely include services such as HIV care, more advanced contraception methods, for example tubal ligation, cervical cancer screening and treatment, or termination of pregnancy (TOP) [17, 18]. These services require highly skilled staff or specialised equipment, and second-line general health services might therefore be better suited. They also might not reach FSWs who only occasionally engage in commercial sex or who do not consider themselves as sex workers and therefore avoid targeted services [12, 22]. General health services might be a better option for these women. Finally, general health services have a more extended network of clinics and a better potential to achieve full geographical coverage. A combination of both targeted and general health services might thus be necessary. The ‘Diagonal Interventions to Fast-Forward Reproductive Health’ (DIFFER) project tested this approach in four cities where sex work is common: Mysore in India, Durban in South Africa, Mombasa in Kenya and Tete in Mozambique [23]. This paper presents findings from the Mozambique site. Results of the other sites are available in the DIFFER final evaluation report [24].

For the overall design of the project, we applied a methodological framework for health systems research developed by Grodos and Mercenier [25]. In this framework, an intervention is designed based on the conceptual model, pre-existing knowledge, and a thorough policy and situational analysis. The intervention is then piloted and its action process and action results are evaluated. For the evaluation, we used an implementation research approach, based on the Consolidated Framework For Implementation Research [26]. We measured implementation outcomes following the WHO framework for implementation research [27]. We assessed several provider-related outcomes: (1) two aspects of feasibility, the degree to which the intervention was implemented as it had been designed (fidelity) and the extent to which it theoretically could be carried out in a Mozambican setting (theoretical feasibility), (2) the perception among providers, managers and policy-makers that the intervention is agreeable (acceptability), and (3) the potential to maintain, institutionalise and expand the intervention (potential sustainability/scalability). In a separate analysis we assessed the intervention’s acceptability and effectiveness from the perspective of the beneficiaries. The results of these beneficiary-related outcomes have been published in a separate paper [28].

## Methods

### Contextual background

The DIFFER project site in Mozambique was situated in an urban area comprising the adjoining cities of Tete and Moatize. Sex work is common due to the presence of a large mining industry and major transport routes intersecting the area. About two thirds of the FSWs are of foreign, mostly Zimbabwean, origin [29]. The baseline policy and situational analysis showed an HIV prevalence of 62%, high rates of genital complaints, unintended pregnancies and sexual violence, and low level of community empowerment [30]. The public health sector was the main provider of SRH care for FSWs, but the services had not been adapted to the needs of that population [31]. FSW-targeted services were available at a stand-alone drop-in clinic (the ‘Night Clinic’) that offered peer outreach and some SRH services (information, condoms, STI treatment, family planning and HIV testing). It was jointly operated by an NGO and the District Health Department. It was located in Moatize and did not sufficiently reach FSWs residing in Tete City, situated 20 km from the clinic.

Following the situational analysis, a context-specific, intervention was designed, applying our diagonal approach, as described in detail elsewhere [32] and summarised in Table 2. In brief, the vertical component entailed expanding the targeted services to cover the entire area and offer a more complete package of SRH services. A mining company was to construct a second stand-alone clinic in Tete City, through a public-private partnership; additional services were to be offered at the clinics; and the peer outreach was to be strengthened and expanded by recruiting and training additional peer outreach workers, both of Mozambican and Zimbabwean origin. Mobilisation of the FSW community comprised capacity building and empowerment through the establishment of a FSW community-based organisation and exchange visits with FSW-organisations elsewhere. In the horizontal component, public health services were to be made more FSW-friendly by sensitizing and training the SRH care providers and appointing one of them as FSW-point of contact at four health centres located near FSW hotspots. These points of contact served as the contact person for FSW-related issues and regularly met with FSW representatives. Linkages between the targeted and general SRH services were to be strengthened by establishing referral systems between the Night Clinics, the peer outreach, and the public health services, and by having regular joint meetings.

The intervention was gradually implemented from mid-2014 onwards. At the end of 2015, the intervention was evaluated. We applied a convergent parallel mixed-methods design [33], combining the analysis of the implementation process with semi-structured interviews with key informants, structured interviews with the health facility points of contact and group discussions with peer outreach workers.

### Analysis of the implementation process

To assess fidelity, we developed a structured digital register (MS Excel 2016), divided by type of activity, in which the details of all activities conducted during implementation were periodically recorded. These included the new services and systems introduced, people recruited, trainings conducted, supplies procured and meetings held. At the end of the intervention, the recorded information was cross-checked and tabulated with the activities that had been planned, to assess what activities had not been executed as planned and why.

### Semi-structured interviews with key informants

The eligibility criterion for participation in the key informant interviews was to have an important role in defining or influencing SRH policies for key populations at national level, or to play an important role in the management of SRH programmes at provincial or district level. We developed a semi-structured guide in English and Portuguese. We explored theoretical feasibility by inquiring about legal or formal barriers to the intervention’s implementation; acceptability by assessing coherence with national guidelines and personal endorsement of the intervention by the informant; and potential sustainability/scalability by asking the respondent’s view on maintaining, institutionalising and expanding the intervention on a national scale.

We interviewed 14 key informants. Six were policy makers or decision takers at national level: two government employees and four representatives of international development agencies or NGOs. At local level, we interviewed two provincial-level and two district-level government officials, two community representatives, and two representatives of a non-governmental agency. The interviews were audio-recorded, transcribed and thematically analysed using N-Vivo 11 by two independent researchers. Data were first deductively and selectively coded according pre-defined themes [34]: outcomes (feasibility, acceptability, sustainability, scalability), how the outcome was evaluated overall (positive or negative), and intervention components (peer outreach, community mobilisation, targeted clinical services, public health services, linkages). The codes were then analysed through cross-tabulation in matrices. We identified differences in views by type of informant (local vs. central; government vs. international organisation).

### Structured interviews with FSW-points of contact

Using a structured questionnaire, we interviewed each of the four FSW-points of contact at the public health facilities. The questionnaire enquired whether the activities to make the services more FSW-friendly carried out at their facility had been practical to implement (fidelity), were agreeable to the providers (acceptability), and their perception on maintaining the intervention (potential sustainability/scalability). The results were transcribed in a spreadsheet (MS Excel 2016) and qualitatively analysed, applying an axial matrix with the three implementation outcomes on the Y-axis and the evaluation of the outcome (positive or negative) on the X-axis.

### Group discussions with peer outreach workers

We held two group discussions, one with nine Mozambican, and one with seven Zimbabwean peer outreach workers, using a semi-structured guide in either Portuguese or English. The guide explored if the peer education activities had been practical to implement and the peer educators’ perceptions on the adequacy and relevance of the conducted activities. The discussions were audio-recorded, transcribed, deductively and selectively coded and thematically analysed using N-Vivo 11 by two independent researchers. The codes were cross-tabulated in matrices by outcome (feasibility, acceptability) and how it was evaluated (positive, negative).

### Mixed analysis

The results of all methods were tabulated, applying a joint display strategy [33], with in the X-axis the implementation outcomes and in the Y-axis the research components (Table 1). We then formulated integrated conclusions, drawing on all sources.

**Table 1:** Mixed-methods analysis matrix: Analysed themes by implementation outcome and research component

		Implementation outcome and themes			
Research component	Number in interview	Fidelity	Theoretical feasibility	Acceptability	Potential sustainability/ scalability
Analysis of the implementation process	–	Extent to which planned activities were conducted	–	–	–
Key informant interviews	14	–	Barriers to implementation	Coherence with national guidelines Endorsement by policy makers and health managers	Potential to maintain, institutionalise and expand on a national scale
FSW-point of contact interviews	4	Extent to which the activities were practical to implement	–	Agreeability of the intervention to the providers	Potential to maintain the intervention
Peer educator group discussions	16		–		–

## Results

### Fidelity and theoretical feasibility

Table 2 presents the comparison of the planned with the implemented activities. Although the intervention had been designed to be practicable with a limited budget, adapted to a low-resource setting, in practice it was not. Many of the planned intervention components could not be fully implemented. The most important challenge to fidelity was the non-establishment of a second targeted clinic in Tete City, which was replaced by mobile clinical outreach, and the expansion of the range of services offered at the Night Clinic and of the peer outreach activities. The major factor was that the financial resources and institutional capacity needed to establish and operate such an intervention were underestimated. In particular, the expansion of the FSW-targeted services required much more resources than were available. Another factor was that some commitments made by partners were not fulfilled. For the construction of the second night clinic, the project opted for a private-public partnership, but in practice this did not work as both the private mining company and the local government were unable to provide adequate support.

**Table 2:** Comparison of the planned and implemented activities (fidelity)

	Planned activities	Progress by the end of the project
*Targeted peer outreach and community mobilisation*	Expand No. of FSW peer educators (PE) from 15 to 30	Partially done. The PE cadre was expanded to 18.
	Orient PEs through a comprehensive training program that comprises the essential information on all SRH components, techniques on how to provide peer education services, and how to use monitoring tools	Mostly done. Two trainings were conducted, one on human rights and empowerment, and one on refreshment of peer education and mobilisation strategies. In addition, 10 new peer educators were trained.
	PEs will be paid a stipend of 1500 MZN (USD35) per month working daily from 4 pm to 10 pm operating from the Night Clinic	Done
	PEs will: • provide essential IEC on all key SRH aspects • distribute free male and female condoms and lubricants • provide information and sensitisation on a correct use of SRH services • implement a system of referral slips • track FSWs who dropped out of certain services, such as HIV care • provide IEC on substance/alcohol abuse and mental health services	Mostly done. Tracking of HIV care defaulters not done.
	PEs will mobilize the community at large to sensitise them about the needs of sex workers to reduce stigma and discrimination	Mostly Done. ICRH-Mozambique conducted sensitisation activities, with involvement of peer educators.
	ICRH-Mozambique will facilitate the creation of a local sex worker association and build capacity among FSWs through workshops and other means	Mostly done. An informal association was created. Capacity was built through exchange visits in India, Malawi and elsewhere in Mozambique.
	Support groups and safe spaces will be encouraged by the project to provide an opportunity and platform for sex workers to discuss and share experiences	Mostly done. The Night Clinic functions as a sort of safe place, a Vulnerable Women’s Support Group was created.
*Targeted clinical services*	The package of services at the Night Clinic will be expanded to include: • IEC on all sexual and reproductive health topics • Provision of male and female condoms and lubricants • Syphilis screening • HIV Testing & Counselling • Free contraception, including long-lasting methods, such as implants, and emergency contraception • Care for incomplete abortions, and support to women with unwanted pregnancies • Sexual and gender-based violence (SGBV) counselling • initiate HIV care, including antiretroviral therapy	Partially done. Female condoms and lubricants were added to the package, emergency contraception is offered, implants are offered but with frequent stock-outs, care for incomplete abortions and the initiation of HIV care were not done, and the SGBV services were only provided for part of the intervention period.
	Memoranda of Understanding will be developed with the district health departments that will describe the responsibilities of each	Done
	In addition to the current Night Clinic in Moatize, a second Night Clinic will be constructed within the City of Tete, offering the same services	Not done. Was replaced by organising mobile clinical outreach.
	FSWs will be invited for routine clinic visits for regular HIV and syphilis testing, genital exams and counselling around e condom use and risk reduction	Done, but limited effectiveness because very few FSWs returned for their follow-up visits.
	HIV+ FSWs will be linked to ART adherence support groups	Not done
*Improve access to the general health services*	Workshops with health facility managers and key SRH providers of 4 selected public health facilities	Done. But late in the project.
	Appointment of FSW points of contact at 4 selected public health facilities	Done. But late in the project.
	Assess whether data on the number of FSW attending the services can be collected in a confidential manner	Done. But late in the project.
	The project will evaluate with the provincial and district health departments if FSWs can be targeted through existing organised outreach activities, such as HIV testing & counselling	Partially done. No FSW-targeted outreach was done by the government, but outreach was done by NGO instead.
	The project will coordinate with the provincial and district health departments and MSF how ART adherence support groups can be further expanded. The support groups will be linked to the Night Clinic and the community mobilisation activities	Not done
*Linkages and referral systems*	Identifying 2 focal persons at each of the 4 health facilities who will be the point of contact	Done. But late in the project.
	Regular meetings between members of the FSW community, the focal persons and health managers of the 4 selected public health facilities, the Night Clinic staff and ICRH-Mozambique	Partially done. There were 7 meetings between all points of contact, the ICRH-Mozambique staff and the peer educators, but no health facility specific meetings between the points of contact and FSW representatives
	Referral and counter-referral systems between the Night Clinics, the 4 health centres and the provincial hospital	Done
	Referral and counter-referral systems between the PEs and the health services	Done
	Tracking of defaulters by PEs	Not done
*Monitoring systems*	The monitoring tools for peer outreach will be adapted and expanded	Done. But late in the project.
	The daily registers will be replaced by an electronic FSW individual monitoring system	Done. But late in the project.
	A system will be developed to monitor attendance by FSWs at the 4 public health facilities	Done. But late in the project.

The consulted policy makers and health managers judged the designed intervention to be theoretically feasible, and saw no legal or other impediments. However, this was conditional on the necessary resources being available.

*‘The experience shows that (the activities) are feasible as long as you have the resources. I mean human resources as well as material resources. It is feasible, yes, but you need the resources: human, financial and material’* (Local-level health manager)

The four health facility points of contact said that the intervention to make the facilities more FSW-friendly had been practicable to implement and that there had been no resistance from the SRH providers. The only reported problem was that FSWs continued not to disclose their occupation when visiting the facility. The peer outreach workers agreed that the peer outreach activities had been practicable, although they faced challenges, such as stigmatisation by the general community, difficulties in reaching certain FSW-subpopulations and the FSWs’ high mobility.

Triangulating the results of the four data sources, we conclude that it was not possible to implement the intervention as designed, but that it is theoretically feasible to implement it in a Mozambican context, if sufficient financial resources are available, institutional capacity is created and all stakeholders fulfil their commitments.

### Acceptability

All informants felt that it is very appropriate to have interventions to improve FSWs’ access to services. It was said to respond to a real need and to be consistent with both the country’s constitution and national health policies.

*‘It is necessary, essential to have this type of services, particularly in areas with the greatest need.’* (National-level stakeholder)

Nevertheless, three key informants, all from international agencies, questioned if all governmental policy makers were genuinely committed to the development of programmes for key populations, such as sex workers and men who have sex with men, and attributed the delays in the operationalisation of the national guidelines to persisting resistance.

*‘For example, we are still awaiting the national guidelines for this population, that until now haven’t been signed yet, and this shows us once more a certain reluctance by the government authorities to accept an investment and a special attention for this population…’* (Local-level stakeholder)

Most intervention components were judged appropriate by informants. All, including the health facility points of contact, were in favour of making the public health services more FSW-friendly and highly appreciated the trainings held and the introduction of points of contact. It was said to be in full agreement with the guidelines that the Ministry of Health (MoH) was developing at the time to make selected health facilities more key population-friendly [35].

*‘We have to have (FSW-)friendly services so that those people come to the health facility and feel comfortable with the offered services, and so that the health care provider as well is at ease in working with those people without stigma, without discrimination’* (National-level policy maker)

Also, peer outreach and community mobilisation were considered appropriate and aligned with national health policies. There was no national strategy yet on these components, but the National AIDS Council had started to develop guidelines on peer outreach.

The only contested intervention component was the clinical services, targeted at a specific population. Because the MoH was in the process of adopting a strategy to improve access to selected public health facilities, three respondents from the central level, two from the government and one from an international agency, no longer saw a need to maintain targeted clinical services. Existing targeted clinics were expected to be temporary until full expansion of FSW-friendly services was achieved. The arguments against targeted services were mostly the perceived low sustainability and cost-effectiveness, the potential stigmatisation of service users, and the principle that the public health system should provide services to all.

*‘The only aspect that falls out, that is not in accordance with our guidelines, is the establishment of night clinics. It wouldn’t be sustainable for the system to have some services that operate at night clinics. And also, in terms of stigmatisation, it is clear that all that is vertical...[meaning at parallel clinics] considering our context it is preferable to follow the path of integration’* (National-level policy maker)

Resistance was lower among representatives of donor and non-governmental agencies of whom three had doubts about the effectiveness of an approach focusing on public health services only. The omission of targeted outreach services in the government guidelines was considered a weakness.

*‘FSWs, it makes a lot of sense to have clinics (such) as these, specifically for this type of groups. These women have a complicated time schedule, some work at night and during daytime they rest and perform other tasks, which makes it difficult for them to go to a health centre… The big problem really is how can these services be adopted by the government.’* (National-level non-governmental stakeholder)

Also at the local level, the three district and provincial health managers, and the community representatives were all in favour of maintaining the Night Clinic and the targeted outreach services.

*‘To have the Night Clinic helps in the sense of having a point of care, very near to the place where high-risk people concentrate, who might be in need of these services and can have them nearby… So, I think it is positive… I think it is a good strategy and it should be given continuity.’* (Local-level health manager)

Our integrated conclusion is that there was a broad consensus on the need to ensure adequate access to SRH services for a key population such as FSWs, but that there was less agreement on how this should be achieved. In particular the approach of having FSW-targeted clinical services is no longer endorsed by the national government.

### Potential sustainability and scalability

All interviewees considered the piloted diagonal model as a whole not sustainable, due to its dependence on international funding. In particular, respondents believed that the government would never absorb the targeted clinical services into its services. Eleven of the 14 respondents also held that the peer outreach component was not sustainable: the MoH did not see it as one of its responsibilities to implement or coordinate peer outreach activities. The National AIDS Council has the responsibility to coordinate community-level HIV interventions, but not to implement or provide significant funding. The expectation was that NGOs would conduct this type of activities with external funding, and they remained therefore highly donor-dependant.

*‘The question of sustainability (of the peer outreach component)... if we look at the financial side, it is difficult to say ‘yes’, because who will sustain it? We know that the responsible institution, which would be the National AIDS Council, is a coordinating organ. It is not an implementing agency. So, who will implement?... They need materials, who will buy these materials?’* (National-level non-governmental stakeholder)

Three informants, all from international agencies, also doubted if there was sufficient institutional capacity within the public health services to maintain the activities to make public health facilities more FSW-friendly without external support.

*‘In the Geração Biz project [a project of adolescent-friendly services] there was technical assistance in each province, in all provincial health departments, that implemented the programme. But when the technical assistance was removed, the programme died. There was no plan to transform this technical assistance in something…’* (National-level non-governmental stakeholder)

Because of the lack of national government endorsement, all informants agreed that the concept of the Night Clinic could not be replicated elsewhere in the country. Regarding other components that were in line with the government policies, respondents said that they should be scaled up nationwide. In particular, the peer outreach model applied by the project and the concept of FSW points of contact, were mentioned by respectively six and five informants, from both the government and international agencies.

The integrated conclusion on potential sustainability is that the piloted model is not fully sustainable, nor replicable, because of lack of government endorsement for targeted clinical services, viewing the provision of community activities as merely a responsibility of civil society, and insufficient long-term financial commitments and institutional capacity.

## Discussion

We designed, piloted and tested an intervention to enhance access to SRH commodities and services by FSWs using an implementation research design. This paper presents the providers’ perspectives on how practicable it had been to implement the activities that were identified as necessary during the situational analysis (fidelity) and if there were structural barriers to implementation, or if the intervention could theoretically be carried out in a Mozambican setting (theoretical feasibility). We further evaluated acceptability by assessing if the intervention was in accordance with national guidelines and endorsed by policy makers, health managers and providers; and if it could be sustained and scaled up over a longer period of time (potential sustainability/scalability).

Our findings suggest that the intervention may not to be entirely feasible nor sustainable or scalable in the Mozambican context. In particular the vertical, targeted component was challenged by a lack of resources and political endorsement. Models of providing targeted services to FSWs, similar to the one planned in our project, have proven to be feasible and sustainable elsewhere [36, 37]. This indicates that if there is a will to mobilise the required long-term resources such interventions are possible, and that the main barrier in Mozambique is the strategic choice made by the government. During the baseline situational analysis, national-level government officials had already expressed a preference to make public health services accessible to all over parallel targeted services [31]. During the course of the intervention, the opposition of national level policy makers to targeted clinical services grew firmer, in particular towards parallel clinics. Targeted outreach offering peer education, condom distribution and HIV testing by NGOs is endorsed by the government, but not directly supported. This stance was not evidence-based, but rather motivated by the argument that it is the government’s duty to provide health services to all and the perceived higher cost-effectiveness of integrated services. Resistance towards a parallel clinic for key populations was markedly lower among government health managers at peripheral level. In contrast with the political perspectives of national leaders, they took a more practical viewpoint, having experienced the advantages of having such clinic. Endorsement was also higher among representatives of international agencies. Their main concern was to ensure adequate access to services, and not necessarily through the public health system.

To date, there is no clear evidence that making general health services FSW-friendly effectively results in increased uptake, and that they are a valid alternative to targeted services [15]. This is supported by the effectiveness analysis of our intervention that demonstrated an increased uptake of services, but that this was almost entirely due to the expansion of the targeted services [28]. In particular the FSW-targeted mobile clinical outreach had raised access to care. Despite having made the public services more FSW-friendly, their utilisation had not (yet) increased.

The perceived high cost of targeted clinical services is at odds with a previous assessment of the Night Clinic that showed that the costs were relatively low in comparison to the number of visits [29]. Targeted interventions have shown to be cost-effective elsewhere [38]. Clearly, more evidence is needed on the effectiveness of making public health facilities FSW-friendly. While rolling out the recently developed strategy of key population-friendly public health facilities, the Mozambican government should carefully monitor and assess its effect. The concept of FSW-points of contact could be integrated into that strategy. Meanwhile, the parallel, targeted services should be maintained wherever possible, and even further developed. Resources need to be provided by the international community, and institutional capacity for quality service delivery further developed. For this to be feasible, key population-targeted clinical services, either through outreach or at parallel clinics, have to be accepted or tolerated by the government. A cost-effectiveness analysis of the targeted component of our intervention could provide additional arguments to substantiate this approach.

Feasibility and sustainability were not only questionable for the targeted clinical services, but also for some other components. Even though all respondents considered peer outreach to be a useful and necessary activity, the government had no intentions to provide funds for this. It fell to civil society organisations to take the initiative, establish, operate and manage this type of activity, with external funding. This typically leads to small-scale, temporary projects that may never have the desired long-term impact. Peer outreach programmes have been demonstrated elsewhere to achieve high coverage and be sustainable, if a comprehensive and budgeted strategy is available [39, 40], and such a strategy is currently lacking in Mozambique.

Even the sustainability of the strategy to create key population-friendly health facilities was not guaranteed. It did not have a budgeted implementation plan and its operationalisation appeared highly dependent on initiatives by non-governmental and international actors. As was mentioned by some informants, previous experiences with youth-friendly services in the country demonstrated that having a strategy is not sufficient to guarantee long-term sustainability [41].

## Conclusions

A ‘diagonal’ intervention to enhance uptake of SRH services by FSWs in Mozambique is currently not fully feasible, sustainable nor replicable, because of insufficient political will and resources for the targeted components. Policy makers need to be aware that until the approach of making general health services more FSW-friendly has proven to be effective, targeted clinical services need to be maintained. A strong targeted peer outreach and community mobilisation component will continue to be necessary in order to educate, sensitise and empower FSWs, and long-term funding needs to be secured.

## Abbreviations

AIDS: Acquired immune-deficiency syndrome
ART: Antiretroviral therapy
DIFFER: Diagonal interventions to fast-forward expanded reproductive health
FSW: Female sex worker
HIV: Human immunodeficiency virus
HPV: Human papilloma virus
ICRH: International centre for reproductive health
IEC: Information, education and communication
MoH: Ministry of health
MSF: Médecins sans frontières
MZN: Mozambican metical
NGO: Non-Governmental Organisation
PE: Peer educator
SGBV: Sexual and gender-based violence
SRH: Sexual and reproductive health
STI: Sexually transmitted infections
